# Cortical Resonance to Visible and Invisible Visual Rhythms

**DOI:** 10.3390/brainsci10010037

**Published:** 2020-01-09

**Authors:** Claudio de’Sperati

**Affiliations:** 1 Laboratory of Action, Perception and Cognition, Vita-Salute San Raffaele University, 20132-Milan, Italy; desperati.claudio@unisr.it; 2 Experimental Psychology Unit, Division of Neuroscience, IRCCS San Raffaele, 20132-Milan, Italy

**Keywords:** perception, video, visual motion, speed, cortex, rhythm, entrainment

## Abstract

Humans are rather poor in judging the right speed of video scenes. For example, a soccer match may be sped up so as to last only 80 min without observers noticing it. However, both adults and children seem to have a systematic, though often biased, notion of what should be the right speed of a given video scene. We therefore explored cortical responsiveness to video speed manipulations in search of possible differences between explicit and implicit speed processing. We applied sinusoidal speed modulations to a video clip depicting a naturalistic scene as well as a traditional laboratory visual stimulus (random dot kinematogram, RDK), and measured both perceptual sensitivity and cortical responses (steady-state visual evoked potentials, SSVEPs) to speed modulations. In five observers, we found a clear perceptual sensitivity increase and a moderate SSVEP amplitude increase with increasing speed modulation strength. Cortical responses were also found with weak, undetected speed modulations. These preliminary findings suggest that the cortex responds globally to periodic video speed modulations, even when observers do not notice them. This entrainment mechanism may be the basis of automatic resonance to the rhythms of the external world.

## 1. Introduction

The capability of judging the correct speed of a dynamic scene in a video clip is surprisingly poor. We have recently shown that (i) speeding up a soccer match video by as much as 12% goes completely undetected [1]; (ii) there are systematic biases in judging the correct video speed, often consisting of speed underestimation [2]; and (iii) 6–7-year-old children judge videos to be slower, as compared to older children and adults [3]. Thus, it appears that there is a mechanism in the brain that implicitly codes a subjective “right” speed of events. This mechanism seems to be specific to event speed, as judgments of video clip speed and video clip duration are not correlated [2].

These findings led us to ask how the speed of a complex video scene is coded in the brain. Taking a somewhat different approach as compared to existing work on visual speed processing [4,5], in this exploratory study we addressed the capability of detecting speed manipulations of complex visual stimuli, both a naturalistic video clip and a laboratory stimulus (random dot kinematogram, RDK). We also had a more specific aim: given that observers appear to be unaware of even large video speed changes, and yet are apparently capable of providing systematic, though often biased, judgments about video speed, there must be a processing stage that automatically and covertly codes the expected speed given the available contextual cues. This processing stage may involve sensory mechanisms, decisional mechanisms, or both. Here, we searched for possible neural correlates of the former, namely, subliminal speed processing. As an initial step towards a more comprehensive understanding of complex visual speed tuning, we recorded perceptual as well as cortical responses to a simple manipulation of the visual stimuli, namely, sinusoidal speed modulation.

## 2. Materials and Methods

### 2.1. Participants

Five adult participants aged between 21 and 35 (all females) took part in this experiment on a voluntary basis and gave informed consent prior to the beginning of the experiments. They had normal or corrected-to-normal vision and had no history of neurological diseases. This study was conducted in accordance with the principles of the Declaration of Helsinki and the “Comitato Etico San Raffaele”.

### 2.2. Stimuli and Tasks

**Figure 1:**
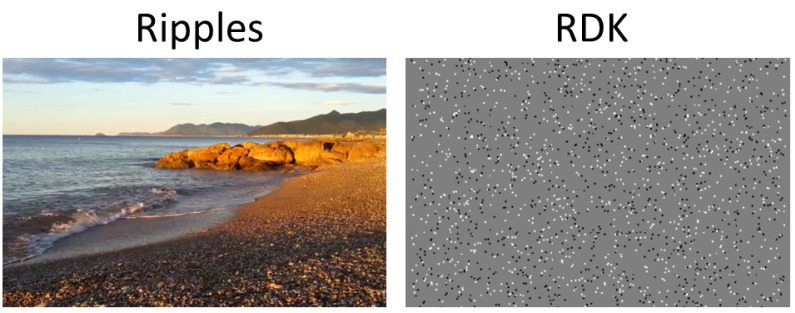
A snapshot of the dynamic visual stimuli used in this study. They were displayed with various sinusoidal speed modulations, which conferred to them a pulsating appearance. In the experiment, there was also a central fixation dot. RDK—random dot kinematogram.

Observers were seated 57 cm in front of a laptop screen. Visual stimuli (Figure 1) consisted of (i) a video clip (1280 × 720 @ 30 Hz) depicting sea ripples on a beach that we had previously used [2]; and (ii) a random dot kinematogram (RDK) (3000 black and white dots, 67 ms lifetime, 5 deg/s maximal speed, 0% coherence). In order to modulate video speed, the frame-rate of the visual stimulus was sinusoidally modulated (frequency, 1, 2, or 4 Hz; amplitude, 10%, 30%, or 50% of the original frame-rate) by controlling in real time the video frame flipping on the graphics board (Nvidia GTX 1060, Santa Clara, CA, USA). As a result, videos acquired a pulsatory rhythm. To ensure uniform conditions in extracting cortical oscillations (see below), in each trial there were 50 speed-modulation cycles regardless of the modulation frequency. Therefore, trials lasted 50, 25, or 12.5 s, respectively, for speed modulations of 1, 2, and 4 Hz. Observers kept their gaze in central fixation and watched the videos while wearing an electroencephalographic (EEG) recording headset (18 trials: 3 frequencies × 3 amplitudes for each visual stimulus, randomly interleaved). They were asked to mentally focus on the pulsation of the visual stimulus. Overall, the session lasted about 20 min.

In a second experimental session, administered on a different day, observers rated in a 9-point scale the perceived strength of the pulsation (108 trials: 3 frequencies × 4 amplitudes × 9 repetitions—including catch trials with zero amplitude, i.e., no speed modulation). Observers were specifically instructed to rate the perceived intensity (strength) of sinusoidal speed modulation. Phenomenally, such rhythmic speed modulation appeared as a pulsation, periodically speeding up and slowing down the stimulus at the experimentally imposed frequency (i.e., 1, 2 or 4 Hz). Stimuli and other conditions were the same as in the first session, except that observers could respond whenever they wished. Overall, the session lasted about 30 min.

### 2.3. EEG Recordings

Scalp electrical activity was recorded through an Enobio device (Neuroelectrics; eight channels, O1, O2, P3, P4, C1, C2, F3, F4; sampling frequency 500 Hz), using the right ear lobe as reference. Electrodes were gel-based passive plates (Ag/AgCl coated; impedance <5 kΩ) and were placed on the scalp by means of an EEG cap. EEG traces were band-pass filtered (0.1–100 Hz).

### 2.4. Data Analyses

To quantify perceptual responses, we used the subjective estimates of video speed modulation strength (pulsation rating). By separating null (rating = 0) and non-null (rating > 0) responses, we transformed gradual judgments into dichotomous yes/no responses, and we applied a signal detection analysis [6]. Basically, the rating task was treated as a multiple yes/no detection task [7], from which we calculated hit rate (non-null responses in signal trials, i.e., trials in which speed was modulated) and false alarm rate (non-null responses in noise trials, i.e., trials in which speed was not modulated). A correction for extreme values was applied [8]. Given the ten-point rating scale used in this experiment, there were nine possible pairs of hits and false alarms: ratings greater than 0 were first considered to be “yes” responses, while a 0 rating was considered to be a “no” response; next, ratings greater than 1 were considered to be “yes” responses, while ratings less than 2 were considered to be “no” responses, and so on, until encompassing all nine pairs of hits and false alarms. A receiver operating characteristic (ROC) curve was thus fitted and the area under the curve (AUC) computed. For each observer, and for each amplitude and frequency of the two stimuli, the perceptual sensitivity to video speed modulation was obtained by converting the AUC into a corresponding d’ index [6].

To quantify cortical responses, we used the Eeglab software ERP averaging tool pop_averager [9] to obtain the averaged traces of each channel over 1-s time windows. Each averaged trace was then fitted to a sinusoidal model (through the Matlab fit function) to compute the cortical response strength, measured as the peak amplitude of the fitted function.

Both the perceptual and the cortical responses were subjected to generalized linear mixed models analysis (GLMM, with diagonal covariance pattern, normal distribution and identity link). The frequency (Freq) and amplitude (Ampl) of video speed modulation were fixed factors, while the video clip (Clip) and participant (Subj) were modeled as random intercept terms to reduce overfitting [10]. Following [11], the recording channel (Chan) for the EEG analysis was also modeled as a random intercept term. The dependent variables were either perceptual sensitivity (PS) or the amplitude of the fitted sinusoidal function (FA). The models were thus “PS ~ 1 + Freq * Ampl + (1 | Clip) + (1 | Subj)” and “FA ~ 1 + Freq * Ampl + (1 | Clip) + (1 | Subj) + (1 | Chan)”, respectively, for perceptual and cortical data.

## 3. Results

**Figure 2:**
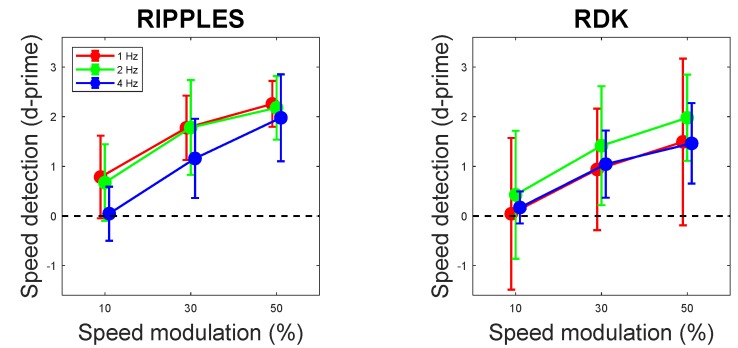
Perceptual sensitivity to video speed modulation as a function of video speed modulation amplitude (10%, 30%, and 50%) and frequency (1, 2, and 4 Hz), shown separately for Ripples and RDK videos. Error bars are 95% confidence intervals.

Observers’ capability of detecting video speed modulation is shown in Figure 2. With weak signals (10% amplitude of video speed modulation), perceptual sensitivity to speed modulation was practically null with both Ripples and RDK video clips (confidence intervals crossed zero in all cases), but increased significantly with speed modulation amplitude. Neither speed modulation frequency nor the interaction frequency × amplitude reached statistical significance (Table 1).

**Figure 3:**
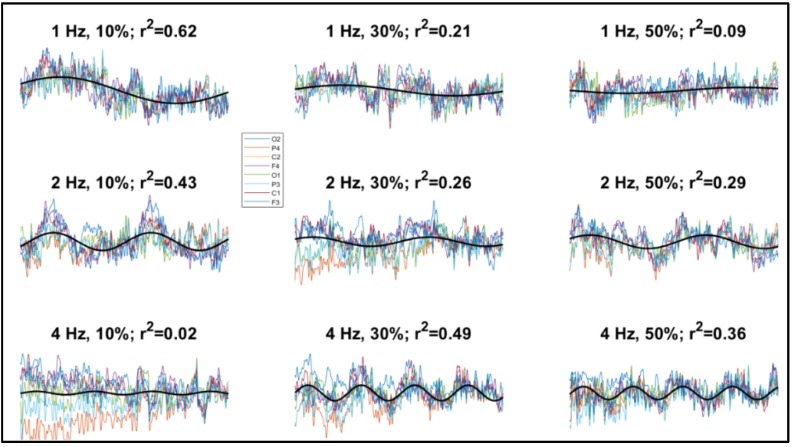
Examples of electroencephalographic (EEG) recordings from one participant showing the traces from all channels (shown in different colors, see legend), averaged across a 1-s time window and superimposed. The sinusoidal best-fitting curves are also shown (black lines; for graphical simplicity, here only a single curve averaged across channels is shown). Also reported are the values of stimulus modulation frequency, stimulus modulation strength, and adjusted R^2^ of the fitted functions.

**Figure 4:**
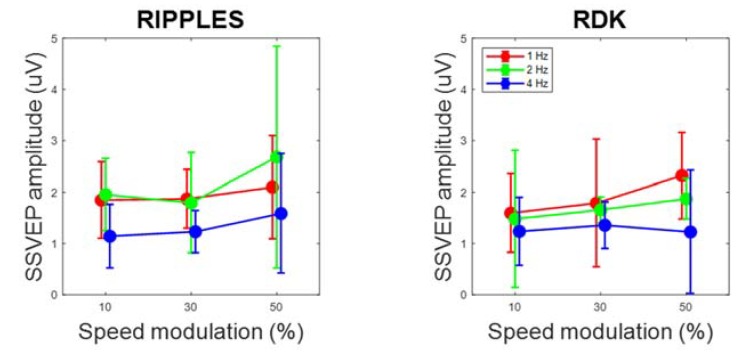
Steady-state visual evoked potential (SSVEP) amplitude as a function of speed modulation amplitude and frequency, shown separately for Ripples and RDK videos. Data have been averaged across participants. Error bars are 95% confidence intervals.

By contrast, cortical oscillatory responses (steady-state visual evoked potentials, SSVEPs) were found at all video speed modulation amplitudes, with mean adjusted R^2^ for the sinusoidal fittings ranging from 13% to 36%, which indicated that a relevant component of cortical activity was pulsating at the stimulus frequency. Although an evident oscillation could not be discerned in all traces (e.g., the 4 Hz, 10% condition of Figure 3), in many cases the cortical potentials followed the rhythm of the video speed (e.g., the 2 Hz, 10% condition of Figure 3). SSVEP amplitude, as computed through sinusoidal fitting, was significantly above zero at all amplitudes and frequencies of speed modulation, as shown by confidence intervals (Figure 4). Also, SSVEP amplitude tended to moderately but significantly increase as speed modulation amplitude increased, though not as steeply as perceptual sensitivity. Speed modulation frequency, but not the interaction frequency × amplitude, also reached statistical significance. The results of these analyses are reported in Table 2. Note also that Figure 3 suggests little SSVEP differentiation across the cortex: indeed, there was a large overlap among the random coefficients of the eight recording channels, with the highest contribution to SSVEP variability coming from F4 (Table 3).

In Table 4, the effect sizes for both perceptual and cortical responses are reported. Perceptual responses tended to be more affected by speed modulation amplitude than speed modulation frequency, whereas the opposite held for cortical responses.

## 4. Discussion

The present study provided initial evidence for (i) cortical oscillatory responsivity to speed modulation in videos either representing a naturalistic scene (a video clip of ripples on the beach) or RDK (with no directional information), and (ii) dissociation between perceptual and cortical responses, consisting in the presence of cortical responses with the perceptually invisible lowest stimulus strength.

### 4.1. Shape and Distribution of Cortical Responses

In some cases, SSVEPs presented rather smooth, sinusoidal-like signal variations, while in other cases more abrupt changes were observed. In this preliminary study, we took an agnostic position as to the actual shape of the cortical response and used sinusoidal fitting as a simple method to quantify the periodicity of scalp potentials, at the cost of losing the details of the full cortical response. We remark, however, that the choice of using sinusoidal fitting was by no means meant to imply that in our conditions SSVEPs had sinusoidal-like waveforms, as is the case with traditional SSVEP protocols where higher stimulus frequencies are used [12]. Given the relatively low frequency of our stimuli, an alternative way to analyze the data would be to use the traditional event-related approach (e.g., finding the peaks and latencies of the various components), which would require, however, a more thorough and systematic consideration of the actual appearance of this particular speed-related cortical potential, including identifying the relevant components. Future work will allow full characterization of this cortical response.

A somewhat unexpected finding was an apparent lack of clear regional differentiation in the amplitude of cortical responses. Luminance-evoked VEPs, as well as cortical responses to motion onset [13,14,15,16,17], involve mainly posterior cortical areas. Yet, in our study, the coefficients for the random effect of a channel were largely overlapping, with only a single electrode (F4) showing a contribution larger than the other ones. One possible explanation is that the oscillatory rhythm we impressed on video clips results in global cortical entrainment, with several components at play (e.g., sensory, attentional, motor, imaginative). Indeed, the instruction to mentally focus on visual pulsation may have favored such multi-component entrainment, making these responses quite different from typical motion-evoked cortical responses. Strictly speaking, our RDK stimuli were not even motion stimuli but speed stimuli (no luminance or directional information), and rhythm/pulsation was the main distinguishing phenomenal characteristic of both RDK and naturalistic stimuli used in the present study. For this reason, they are likely to induce high-level resonance that may go beyond sensory stimulation (see, e.g., [18] for a similar consideration in the auditory domain).

Alternatively, our approach might simply not have been fine-grained enough to detect small regional differences. It is possible that with a more in-depth approach (e.g., high-density electroencephalography with event-related component analyses and source reconstruction) and a larger sample size, some differences would emerge.

### 4.2. Attentive Subliminal Resonance 

We found diffuse cortical responses with the weakest video speed modulation (10%), a condition that was associated with null perceptual sensitivity. This phenomenon calls for subliminal speed entrainment, indicative of an automatic resonance process, which may qualify as a form of attentive subliminal processing [19], or at least at the fringe of awareness. By further noting the disparity between the steep increase in perceptual sensitivity at increasing speed modulation amplitudes and the corresponding very moderate increase in cortical responsiveness, it is tempting to speculate that, at least for certain phenomena, global cortical activation to visual stimuli may reflect automatic processing without necessarily being the signature of perceptual awareness.

However, especially when null results are obtained under low statistical power, it should be borne in mind that “absence of evidence” rather than “evidence of absence” is the proper underlying notion. Furthermore, when the goal is to measure perceptual awareness, null results can be problematic on their own, as it is often difficult to clearly tease apart null from fringe perception [20]. Perceptual ratings may help in this regard, as they involve more gradual responses rather than gross dichotomous responses [21]. Exploiting all possible response criteria behind perceptual ratings through the ROC curve afforded a reliable index of perceptual awareness [6]. Thus, pending further investigation with a larger sample size (see below), we believe that most stimuli with 10% speed modulation were closer to a condition of null than fringe speed modulation perception.

### 4.3. Limitations

The first limitation of this exploratory study is obviously the small sample size. While for some psychophysical designs this numerosity may be reasonable, to provide good statistical power, and also to address possible individual differences, the number of participants should be increased.

Another limitation is the small number of EEG electrodes. With eight electrodes, we did not even attempt to compute a spatial map of cortical activity. This issue should be addressed with more sophisticated EEG recordings and analyses. Our goal here was simply to provide initial evidence of cortical entrainment.

We should also note a limitation of the experimental design. For practical reasons, we administered the cortical recording session before the perceptual session. This could have introduced a carry-over effect, i.e., observers’ higher sensitivity in the perceptual session as a result of learning. This could be one reason for the steeper rise of perceptual responses with video speed modulation amplitude, as compared to cortical responses. Note, however, that the direction of such carry-over effect, if any (the cortical session being administered before the perceptual session, and not vice-versa), would reinforce the evidence for a dissociation between cortical and perceptual responses: the perceptual responses in the trials with the lowest speed modulation strength were statistically indistinguishable from the responses in the trials without speed modulation, despite the possible beneficial effect of previous exposure to the stimuli.

## Figures and Tables

**Table 1:** Results of the generalized linear mixed models (GLMM) analysis of perceptual sensitivity.

	e	t	d	*p*	l	u
Amplitude	0.036	4.437	86	<0.001	0.020	0.051
Frequency	−0.0136	−1.312	86	0.193	−0.341	0.070
Amplitude:Frequency	0.001	0.402	86	0.689	−0.005	0.007

Video speed modulation amplitude, but not frequency, or their interaction, was statistically significant. Abbreviations: e—coefficient estimate, t—t-statistics (Wald t-test), d—degrees of freedom, *p*—*p*-value, l—lower confidence bound, u—upper confidence bound.

**Table 2:** Results of the GLMM analysis of SSVEP amplitude.

	e	t	d	*p*	l	u
Amplitude	16.439	3.030	716	0.003	5.789	27.090
Frequency	−144.841	−2.068	716	0.039	−282.341	−7.341
Amplitude:Frequency	−2.542	−1.240	716	0.215	−6.568	1.484

Video speed modulation amplitude and frequency, but not their interaction, were statistically significant. See Table 1 for abbreviations.

**Table 3:** Coefficient estimates resulting from the GLMM analysis of SSVEP amplitude for the eight recording channels. See text and Table 1 for abbreviations.

Channel	e	t	d	*p*	l	u
O1	153.240	1.218	716	0.224	400.210	93.734
O2	85.014	0.676	716	0.499	331.990	161.960
P3	30.566	0.243	716	0.808	277.540	216.410
P4	185.070	1.471	716	0.142	61.900	432.040
C1	141.630	1.126	716	0.261	388.610	105.340
C2	231.030	1.834	716	0.067	478.000	15.944
F3	78.625	0.625	716	0.532	168.350	325.600
F4	377.780	3.003	716	0.003	130.810	624.750

**Table 4:** Estimates of effect size (partial η^2^) of video speed modulation amplitude and frequency on perceptual and cortical responses, shown separately for Ripples and RDK stimuli.

	Perceptual Responses		Cortical Responses	
	Ripples	RDK	Ripples	RDK
Amplitude	0.598	0.649	0.249	0.170
Frequency	0.164	0.172	0.440	0.381
